# 2-Amino-4-(4-chloro­phen­yl)-6-(pyrrolidin-1-yl)pyridine-3,5-dicarbonitrile

**DOI:** 10.1107/S1600536812009051

**Published:** 2012-03-10

**Authors:** S. Antony Inglebert, Jayabal Kamalraja, K. Sethusankar, Gnanasambandam Vasuki

**Affiliations:** a Department of Physics, Sri Ram Engineering College, Chennai 602 024, India; b Department of Chemistry, Pondichery University, Pondichery 605 014, India; c Department of Physics, RKM Vivekananda College (Autonomous), Chennai 600 004, India

## Abstract

In the title compound, C_17_H_14_ClN_5_, two C atoms and their attached H atoms of the pyrrolidine ring are disordered over two sets of sites with an occupancy ratio of 0.638 (10):0.362 (10). The benzene and pyridine rings are inclined to one another by 60.57 (8)°. In the crystal, the amino group forms an N—H⋯N hydrogen bond with one of the cyano groups, linking the mol­ecules into chains along [010].

## Related literature

For a similar compound, see: Inglebert *et al.* (2011 ▶). For related structures, see: Chao *et al.* (1975 ▶); Kvick *et al.* (1976 ▶). For bond-length data, see: Atoji & Lipscomb (1953 ▶). For puckering parameters, see: Cremer & Pople (1975 ▶).
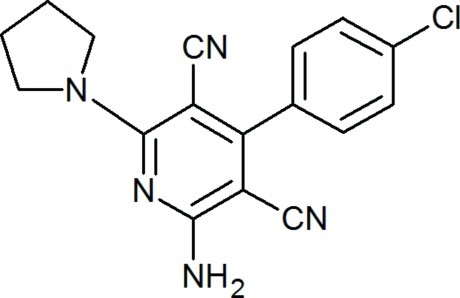


## Experimental

### 

#### Crystal data

C_17_H_14_ClN_5_
*M* *_r_* = 323.77
Triclinic, 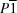

*a* = 7.318 (5) Å
*b* = 9.060 (5) Å
*c* = 12.011 (5) Å
α = 87.196 (5)°
β = 80.477 (5)°
γ = 83.795 (5)°
*V* = 780.4 (8) Å^3^
*Z* = 2
Mo *K*α radiation
μ = 0.25 mm^−1^
*T* = 295 K
0.35 × 0.30 × 0.25 mm

#### Data collection

Bruker Kappa APEXII CCD diffractometer
Absorption correction: multi-scan (*SADABS*; Bruker, 2008 ▶) *T*_min_ = 0.916, *T*_max_ = 0.939
6077 measured reflections
3570 independent reflections
1887 reflections with *I* > 2σ(*I*)
*R*_int_ = 0.027

#### Refinement

*R*[*F* ^2^ > 2σ(*F* ^2^)] = 0.042
*wR*(*F* ^2^) = 0.097
*S* = 0.85
3570 reflections
237 parameters
11 restraints
H atoms treated by a mixture of independent and constrained refinement
Δρ_max_ = 0.20 e Å^−3^
Δρ_min_ = −0.24 e Å^−3^

### 

Data collection: *APEX2* (Bruker, 2008 ▶); cell refinement: *SAINT* (Bruker, 2008 ▶); data reduction: *SAINT*; program(s) used to solve structure: *SHELXS97* (Sheldrick, 2008 ▶); program(s) used to refine structure: *SHELXL97* (Sheldrick, 2008 ▶); molecular graphics: *ORTEP-3* (Farrugia, 1997 ▶); software used to prepare material for publication: *SHELXL97* and *PLATON* (Spek, 2009 ▶).

## Supplementary Material

Crystal structure: contains datablock(s) global, I. DOI: 10.1107/S1600536812009051/rk2335sup1.cif

Structure factors: contains datablock(s) I. DOI: 10.1107/S1600536812009051/rk2335Isup2.hkl

Supplementary material file. DOI: 10.1107/S1600536812009051/rk2335Isup3.cml

Additional supplementary materials:  crystallographic information; 3D view; checkCIF report

## Figures and Tables

**Table 1:** Hydrogen-bond geometry (Å, °)

*D*—H⋯*A*	*D*—H	H⋯*A*	*D*⋯*A*	*D*—H⋯*A*
N2—H2*B*⋯N4^i^	0.92 (1)	2.12 (1)	2.992 (3)	160 (2)

Symmetry code: (i) 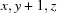.

**Fig. 1.**
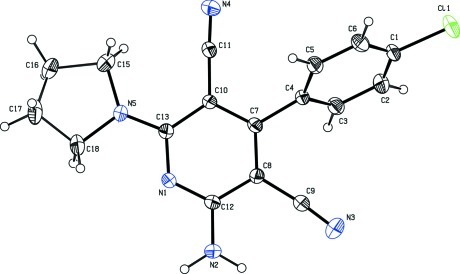
The molecular structure of the title compound, showing the atom numbering scheme. Displacement ellipsoids are drawn at 30% probability level. H atoms are presented as small spheres of arbitary radius. The minor occupancy disordered atoms have been omitted for clarity.

**Fig. 2.**
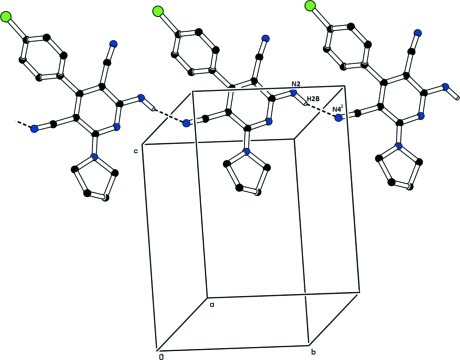
The packing diagram of the title compound, which shows intermolecular N2—H2B···N4i interactions (dashed lines). H atoms not involved in hydrogen bonds have been omitted for clarity.

## supplementary crystallographic information

### Comment

Pyridine and its derivatives play an important role in hetrocyclic chemistry. Pyridine containing compounds are the new class of anti–*HIV* molecules, which particularly inhibit *RNA* dependent *DNA* polymerase or reverse transcriptace and thus act as non–nucleoside reverse transcriptace inhibitors. They also exhibit cytotoxic, anti–cancer, anti–tumour and anti–bacterial activity.

The pyrrolidine ring adopts a twisted conformation in both the major and minor conformers (occupancy factors of 0.638 (10)/0.362 (10) respectively). Puckering parameters (Cremer & Pople, 1975) are q_2_ and φ_2_, of 0.422 (6)Å and 273.9 (4)° for the major conformer (N5/C15/C16/C17/C18) and 0.469 (10)Å and 86.4 (6)°, respectively, for the minor conformer (N5/C15/C16'/C17'/C18).

The bond lengths of the nitrile groups attached to pyridine ring are typical (N4≡C11 = 1.148 (2)Å and C9≡N3 = 1.142 (2)Å). The nitrile groups form angles with parent C atoms: 177.1 (2)° and 174.5 (2)°. The sum angles around the atom C12 are slightly less 360° (real 358.0 (2)°) - deformed by the amino group, as seen in other aminopyridines (Chao *et al.*, 1975; Kvick *et al.*, 1976). This behaviour characterizes the resonance of the N2 lone pair with the aromatic ring. The effect can also be verified by the shortening of the C12—N2 bond (1.345 (2)Å) relative to a normal single C—N bond (1.483Å for C—N in methaneamine (Atoji & Lipscomb, 1953).

The amino group is planar with the pyridine ring as indicated by the torsion angle N2—C12—N1—C13 = 179.75 (16)°. The chlorine atom attached at C1 deviates by -0.0817 (3)Å from the mean plane of the phenyl ring. The title structure exhibits structural similarities with the previously reported structure (Inglebert *et al.*, 2011).

In the crystal structure, the classical intermolecular N2—H2B···N4^i^ hydrogen bonds link the molecules into chains along the *b* axis. Symmetry code: (i) *x*, *y*+1, *z*.

### Experimental

A mixture of 4–chlorobenzaldehyde (2 mmoL, 0.28 g), malononitrile (3 mmoL, 0.198 g), pyrrolidine (1.5 mmoL, 0.1 g) was stirred without any solvent at room temperature. A solid appeared immediately which has dissolved in a minimum amount (3 ml) of ethanol and the solution was refluxed until completion of the reaction (monitered by *TLC*). The reaction mixture was cooled. Ethanol was evaporated under reduced pressure and the residue was extracted with dicholoromethane (3×10 ml). Evaporation of solvent left the crude solid which was subjected to silica gel column chromatography [25:75 ethyl acetate/hexane] and the product was recrysallized from dichloromethane.

### Refinement

H atoms attached to C atoms were positioned geometrically and constrained to ride on their parent atoms, with C—H = 0.93Å (aromatic H) and C—H = 0.97Å (methylene H) *U*_iso_(H) = 1.2*U*_eq_(C). H atoms of amino group were located from difference Fourier map and refined freely.

### Crystal data

**Table:**

C_17_H_14_ClN_5_	*Z* = 2
*M**_r_* = 323.77	*F*(000) = 336
Triclinic, *P*1	*D*_x_ = 1.378 Mg m^−^^3^
Hall symbol: -P 1	Mo *K*α radiation, λ = 0.71073 Å
*a* = 7.318 (5) Å	Cell parameters from 3570 reflections
*b* = 9.060 (5) Å	θ = 2.8–29.3°
*c* = 12.011 (5) Å	µ = 0.25 mm^−^^1^
α = 87.196 (5)°	*T* = 295 K
β = 80.477 (5)°	Block, colourless
γ = 83.795 (5)°	0.35 × 0.30 × 0.25 mm
*V* = 780.4 (8) Å^3^	

### Data collection

**Table:**

Bruker Kappa APEXII CCD diffractometer	3570 independent reflections
Radiation source: fine-focus sealed tube	1887 reflections with *I* > 2σ(*I*)
Graphite monochromator	*R*_int_ = 0.027
ω and φ scans	θ_max_ = 29.3°, θ_min_ = 2.8°
Absorption correction: multi-scan (*SADABS*; Bruker, 2008)	*h* = −9→9
*T*_min_ = 0.916, *T*_max_ = 0.939	*k* = −12→11
6077 measured reflections	*l* = −15→16

### Refinement

**Table:**

Refinement on *F*^2^	Secondary atom site location: difference Fourier map
Least-squares matrix: full	Hydrogen site location: inferred from neighbouring sites
*R*[*F*^2^ > 2σ(*F*^2^)] = 0.042	H atoms treated by a mixture of independent and constrained refinement
*wR*(*F*^2^) = 0.097	*w* = 1/[σ^2^(*F*_o_^2^) + (0.0456*P*)^2^] where *P* = (*F*_o_^2^ + 2*F*_c_^2^)/3
*S* = 0.85	(Δ/σ)_max_ = 0.001
3570 reflections	Δρ_max_ = 0.20 e Å^−^^3^
237 parameters	Δρ_min_ = −0.24 e Å^−^^3^
11 restraints	Extinction correction: *SHELXL97* (Sheldrick, 2008), Fc^*^=kFc[1+0.001xFc^2^λ^3^/sin(2θ)]^-1/4^
Primary atom site location: structure-invariant direct methods	Extinction coefficient: 0.014 (2)

### Special details

**Table:**

Geometry. All s.u.'s (except the s.u. in the dihedral angle between two l.s. planes) are estimated using the full covariance matrix. The cell s.u.'s are taken into account individually in the estimation of s.u.'s in distances, angles and torsion angles; correlations between s.u.'s in cell parameters are only used when they are defined by crystal symmetry. An approximate (isotropic) treatment of cell s.u.'s is used for estimating s.u.'s involving l.s. planes.
Refinement. Refinement of *F*^2^ against ALL reflections. The weighted *R*–factor *wR* and goodness of fit *S* are based on *F*^2^, conventional *R*–factors *R* are based on *F*, with *F* set to zero for negative *F*^2^. The threshold expression of *F*^2^ > σ(*F*^2^) is used only for calculating *R*–factors(gt) *etc*. and is not relevant to the choice of reflections for refinement. *R*–factors based on *F*^2^ are statistically about twice as large as those based on *F*, and *R*–factors based on ALL data will be even larger.

### Fractional atomic coordinates and isotropic or equivalent isotropic displacement parameters (Å^2^)

**Table:**

	*x*	*y*	*z*	*U*_iso_*/*U*_eq_	Occ. (<1)
C1	0.7572 (3)	0.0214 (2)	1.33801 (16)	0.0432 (5)	
C2	0.9078 (3)	0.1010 (2)	1.30733 (17)	0.0459 (5)	
H2	1.0082	0.0866	1.3465	0.055*	
C3	0.9092 (3)	0.2024 (2)	1.21800 (16)	0.0390 (5)	
H3	1.0107	0.2572	1.1977	0.047*	
C4	0.7624 (2)	0.22397 (19)	1.15801 (14)	0.0311 (4)	
C5	0.6137 (3)	0.1396 (2)	1.18885 (16)	0.0409 (5)	
H5	0.5149	0.1510	1.1484	0.049*	
C6	0.6108 (3)	0.0393 (2)	1.27858 (17)	0.0486 (5)	
H6	0.5101	−0.0163	1.2990	0.058*	
C7	0.7579 (2)	0.34351 (18)	1.06767 (15)	0.0287 (4)	
C8	0.7651 (2)	0.48942 (18)	1.09633 (15)	0.0299 (4)	
C9	0.7812 (3)	0.5310 (2)	1.20691 (18)	0.0383 (5)	
C10	0.7429 (2)	0.31384 (18)	0.95678 (14)	0.0288 (4)	
C11	0.7380 (3)	0.1630 (2)	0.93015 (15)	0.0368 (5)	
C12	0.7507 (2)	0.60362 (18)	1.01258 (15)	0.0291 (4)	
C13	0.7366 (2)	0.43534 (19)	0.87573 (15)	0.0297 (4)	
N5	0.7283 (2)	0.42030 (16)	0.76655 (12)	0.0364 (4)	
C18	0.7235 (3)	0.5492 (2)	0.68715 (16)	0.0519 (6)	
H18A	0.8436	0.5884	0.6720	0.062*	
H18B	0.6299	0.6271	0.7177	0.062*	
C17	0.6766 (10)	0.4927 (6)	0.5828 (4)	0.0567 (15)	0.638 (10)
H17A	0.7199	0.5533	0.5169	0.068*	0.638 (10)
H17B	0.5437	0.4872	0.5884	0.068*	0.638 (10)
C16	0.7840 (11)	0.3389 (7)	0.5805 (4)	0.0657 (18)	0.638 (10)
H16A	0.9162	0.3446	0.5558	0.079*	0.638 (10)
H16B	0.7389	0.2747	0.5310	0.079*	0.638 (10)
C17'	0.7780 (18)	0.4694 (13)	0.5745 (7)	0.068 (3)	0.361 (10)
H17C	0.7346	0.5301	0.5135	0.082*	0.362 (10)
H17D	0.9121	0.4476	0.5566	0.082*	0.362 (10)
C16'	0.6834 (18)	0.3273 (12)	0.5927 (8)	0.061 (3)	0.362 (10)
H16C	0.7328	0.2556	0.5352	0.073*	0.362 (10)
H16D	0.5492	0.3453	0.5982	0.073*	0.362 (10)
N1	0.73637 (19)	0.57681 (15)	0.90682 (12)	0.0323 (4)	
N2	0.7503 (2)	0.74643 (18)	1.03834 (16)	0.0440 (4)	
N3	0.7954 (3)	0.5758 (2)	1.29203 (16)	0.0641 (6)	
N4	0.7312 (3)	0.04021 (19)	0.91349 (15)	0.0589 (5)	
C15	0.7437 (4)	0.2824 (2)	0.70643 (18)	0.0634 (7)	
H15A	0.6287	0.2354	0.7221	0.076*	
H15B	0.8451	0.2130	0.7257	0.076*	
Cl1	0.74935 (9)	−0.10029 (6)	1.45478 (5)	0.0728 (2)	
H2A	0.759 (2)	0.771 (2)	1.1096 (10)	0.048 (6)*	
H2B	0.745 (3)	0.8223 (17)	0.9853 (14)	0.066 (7)*	

### Atomic displacement parameters (Å^2^)

**Table:**

	*U*^11^	*U*^22^	*U*^33^	*U*^12^	*U*^13^	*U*^23^
C1	0.0684 (14)	0.0303 (11)	0.0283 (11)	−0.0027 (10)	−0.0039 (10)	0.0067 (9)
C2	0.0557 (13)	0.0439 (12)	0.0387 (12)	−0.0010 (10)	−0.0146 (10)	0.0077 (10)
C3	0.0415 (11)	0.0378 (11)	0.0385 (12)	−0.0088 (9)	−0.0076 (9)	0.0051 (9)
C4	0.0398 (11)	0.0271 (9)	0.0256 (10)	−0.0039 (8)	−0.0034 (8)	0.0015 (8)
C5	0.0487 (12)	0.0400 (11)	0.0370 (12)	−0.0138 (9)	−0.0116 (9)	0.0056 (9)
C6	0.0612 (13)	0.0420 (12)	0.0434 (13)	−0.0218 (10)	−0.0029 (11)	0.0084 (10)
C7	0.0281 (9)	0.0274 (10)	0.0307 (10)	−0.0048 (7)	−0.0050 (8)	0.0030 (8)
C8	0.0330 (10)	0.0300 (10)	0.0270 (10)	−0.0056 (8)	−0.0044 (8)	−0.0009 (8)
C9	0.0478 (12)	0.0321 (11)	0.0352 (12)	−0.0076 (9)	−0.0064 (9)	0.0022 (9)
C10	0.0337 (10)	0.0251 (9)	0.0274 (10)	−0.0044 (8)	−0.0041 (8)	0.0000 (8)
C11	0.0504 (12)	0.0305 (11)	0.0285 (11)	−0.0044 (9)	−0.0044 (9)	0.0035 (8)
C12	0.0288 (10)	0.0254 (10)	0.0329 (11)	−0.0023 (8)	−0.0047 (8)	−0.0007 (8)
C13	0.0306 (10)	0.0300 (10)	0.0287 (11)	−0.0034 (8)	−0.0054 (8)	−0.0006 (8)
N5	0.0507 (10)	0.0339 (9)	0.0257 (9)	−0.0057 (7)	−0.0090 (7)	−0.0005 (7)
C18	0.0719 (15)	0.0521 (13)	0.0318 (12)	−0.0033 (11)	−0.0136 (11)	0.0086 (10)
C17	0.059 (3)	0.083 (3)	0.031 (2)	−0.018 (3)	−0.015 (3)	0.0128 (19)
C16	0.108 (5)	0.064 (3)	0.029 (2)	−0.019 (4)	−0.011 (3)	−0.007 (2)
C17'	0.076 (8)	0.093 (8)	0.041 (5)	−0.027 (7)	−0.019 (5)	0.014 (4)
C16'	0.073 (7)	0.071 (6)	0.038 (4)	−0.009 (5)	−0.006 (5)	−0.008 (4)
N1	0.0412 (9)	0.0268 (8)	0.0297 (9)	−0.0040 (7)	−0.0089 (7)	0.0027 (7)
N2	0.0700 (12)	0.0262 (9)	0.0384 (11)	−0.0060 (8)	−0.0158 (9)	−0.0027 (8)
N3	0.0930 (15)	0.0639 (13)	0.0390 (12)	−0.0137 (11)	−0.0146 (11)	−0.0101 (10)
N4	0.0976 (15)	0.0308 (10)	0.0480 (12)	−0.0093 (10)	−0.0091 (10)	−0.0003 (9)
C15	0.1068 (19)	0.0471 (14)	0.0404 (14)	−0.0073 (13)	−0.0214 (13)	−0.0100 (11)
Cl1	0.1163 (6)	0.0539 (4)	0.0454 (4)	−0.0100 (3)	−0.0114 (3)	0.0240 (3)

### Geometric parameters (Å, º)

**Table:**

C1—C2	1.372 (3)	C13—N1	1.352 (2)
C1—C6	1.374 (3)	N5—C15	1.458 (3)
C1—Cl1	1.7385 (19)	N5—C18	1.472 (2)
C2—C3	1.377 (3)	C18—C17	1.481 (5)
C2—H2	0.9300	C18—C17'	1.538 (8)
C3—C4	1.382 (2)	C18—H18A	0.9700
C3—H3	0.9300	C18—H18B	0.9700
C4—C5	1.388 (2)	C17—C16	1.523 (7)
C4—C7	1.497 (2)	C17—H17A	0.9700
C5—C6	1.374 (3)	C17—H17B	0.9700
C5—H5	0.9300	C16—C15	1.564 (5)
C6—H6	0.9300	C16—H16A	0.9700
C7—C8	1.391 (2)	C16—H16B	0.9700
C7—C10	1.396 (2)	C17'—C16'	1.517 (9)
C8—C12	1.415 (2)	C17'—H17C	0.9700
C8—C9	1.425 (3)	C17'—H17D	0.9700
C9—N3	1.143 (2)	C16'—C15	1.527 (8)
C10—C11	1.424 (3)	C16'—H16C	0.9700
C10—C13	1.435 (2)	C16'—H16D	0.9700
C11—N4	1.147 (2)	N2—H2A	0.907 (9)
C12—N1	1.328 (2)	N2—H2B	0.916 (9)
C12—N2	1.345 (2)	C15—H15A	0.9700
C13—N5	1.337 (2)	C15—H15B	0.9700
			
C2—C1—C6	120.58 (18)	C17'—C18—H18A	87.7
C2—C1—Cl1	119.71 (16)	N5—C18—H18B	110.6
C6—C1—Cl1	119.71 (16)	C17—C18—H18B	110.6
C1—C2—C3	119.43 (18)	C17'—C18—H18B	136.3
C1—C2—H2	120.3	H18A—C18—H18B	108.8
C3—C2—H2	120.3	C18—C17—C16	100.5 (4)
C2—C3—C4	121.07 (18)	C18—C17—H17A	111.7
C2—C3—H3	119.5	C16—C17—H17A	111.7
C4—C3—H3	119.5	C18—C17—H17B	111.7
C3—C4—C5	118.45 (17)	C16—C17—H17B	111.7
C3—C4—C7	120.55 (16)	H17A—C17—H17B	109.4
C5—C4—C7	120.84 (16)	C17—C16—C15	103.0 (4)
C6—C5—C4	120.70 (18)	C17—C16—H16A	111.2
C6—C5—H5	119.7	C15—C16—H16A	111.2
C4—C5—H5	119.7	C17—C16—H16B	111.2
C1—C6—C5	119.75 (19)	C15—C16—H16B	111.2
C1—C6—H6	120.1	H16A—C16—H16B	109.1
C5—C6—H6	120.1	C16'—C17'—C18	105.0 (7)
C8—C7—C10	119.14 (16)	C16'—C17'—H17C	110.7
C8—C7—C4	118.54 (16)	C18—C17'—H17C	110.7
C10—C7—C4	122.30 (16)	C16'—C17'—H17D	110.7
C7—C8—C12	118.67 (16)	C18—C17'—H17D	110.7
C7—C8—C9	123.41 (16)	H17C—C17'—H17D	108.8
C12—C8—C9	117.90 (16)	C17'—C16'—C15	96.3 (7)
N3—C9—C8	174.5 (2)	C17'—C16'—H16C	112.5
C7—C10—C11	117.62 (15)	C15—C16'—H16C	112.5
C7—C10—C13	118.72 (15)	C17'—C16'—H16D	112.5
C11—C10—C13	123.66 (16)	C15—C16'—H16D	112.5
N4—C11—C10	177.1 (2)	H16C—C16'—H16D	110.0
N1—C12—N2	117.00 (16)	C12—N1—C13	119.72 (15)
N1—C12—C8	122.72 (16)	C12—N2—H2A	120.3 (12)
N2—C12—C8	120.28 (17)	C12—N2—H2B	122.1 (13)
N5—C13—N1	114.88 (15)	H2A—N2—H2B	117.5 (18)
N5—C13—C10	124.18 (16)	N5—C15—C16'	105.3 (5)
N1—C13—C10	120.93 (16)	N5—C15—C16	101.7 (3)
C13—N5—C15	127.41 (16)	C16'—C15—C16	27.8 (3)
C13—N5—C18	121.73 (15)	N5—C15—H15A	111.4
C15—N5—C18	110.50 (16)	C16'—C15—H15A	84.8
N5—C18—C17	105.5 (3)	C16—C15—H15A	111.4
N5—C18—C17'	99.8 (5)	N5—C15—H15B	111.4
C17—C18—C17'	28.3 (4)	C16'—C15—H15B	131.2
N5—C18—H18A	110.6	C16—C15—H15B	111.4
C17—C18—H18A	110.6	H15A—C15—H15B	109.3
			
C6—C1—C2—C3	1.8 (3)	C7—C10—C13—N1	−3.1 (2)
Cl1—C1—C2—C3	−176.95 (15)	C11—C10—C13—N1	178.08 (16)
C1—C2—C3—C4	−0.8 (3)	N1—C13—N5—C15	173.81 (18)
C2—C3—C4—C5	−0.8 (3)	C10—C13—N5—C15	−7.1 (3)
C2—C3—C4—C7	174.70 (17)	N1—C13—N5—C18	1.3 (2)
C3—C4—C5—C6	1.4 (3)	C10—C13—N5—C18	−179.57 (17)
C7—C4—C5—C6	−174.06 (17)	C13—N5—C18—C17	−168.9 (3)
C2—C1—C6—C5	−1.2 (3)	C15—N5—C18—C17	17.4 (4)
Cl1—C1—C6—C5	177.55 (15)	C13—N5—C18—C17'	162.6 (5)
C4—C5—C6—C1	−0.5 (3)	C15—N5—C18—C17'	−11.1 (5)
C3—C4—C7—C8	−57.7 (2)	N5—C18—C17—C16	−37.2 (6)
C5—C4—C7—C8	117.6 (2)	C17'—C18—C17—C16	44.7 (9)
C3—C4—C7—C10	123.59 (19)	C18—C17—C16—C15	43.0 (8)
C5—C4—C7—C10	−61.0 (2)	N5—C18—C17'—C16'	37.4 (11)
C10—C7—C8—C12	2.1 (2)	C17—C18—C17'—C16'	−67.1 (10)
C4—C7—C8—C12	−176.63 (16)	C18—C17'—C16'—C15	−47.4 (13)
C10—C7—C8—C9	−179.69 (16)	N2—C12—N1—C13	179.75 (15)
C4—C7—C8—C9	1.6 (3)	C8—C12—N1—C13	−0.5 (2)
C8—C7—C10—C11	179.32 (16)	N5—C13—N1—C12	−177.73 (15)
C4—C7—C10—C11	−2.0 (2)	C10—C13—N1—C12	3.1 (2)
C8—C7—C10—C13	0.4 (2)	C13—N5—C15—C16'	168.2 (5)
C4—C7—C10—C13	179.06 (16)	C18—N5—C15—C16'	−18.6 (5)
C7—C8—C12—N1	−2.2 (2)	C13—N5—C15—C16	−163.4 (3)
C9—C8—C12—N1	179.51 (16)	C18—N5—C15—C16	9.8 (4)
C7—C8—C12—N2	177.60 (17)	C17'—C16'—C15—N5	39.5 (11)
C9—C8—C12—N2	−0.7 (2)	C17'—C16'—C15—C16	−46.5 (9)
C7—C10—C13—N5	177.84 (15)	C17—C16—C15—N5	−32.6 (7)
C11—C10—C13—N5	−1.0 (3)	C17—C16—C15—C16'	68.1 (11)

### Hydrogen-bond geometry (Å, º)

**Table:**

*D*—H···*A*	*D*—H	H···*A*	*D*···*A*	*D*—H···*A*
N2—H2*B*···N4^i^	0.92 (1)	2.12 (1)	2.992 (3)	160 (2)

Symmetry code: (i) *x*, *y*+1, *z*.
